# Genetic Evidences of Biosurfactant Production in Two *Bacillus subtilis* Strains MB415 and MB418 Isolated From Oil Contaminated Soil

**DOI:** 10.3389/fbioe.2022.855762

**Published:** 2022-04-26

**Authors:** Azra Yasmin, Fozia Aslam, Anila Fariq

**Affiliations:** ^1^ Microbiology and Biotechnology Research Lab, Department of Biotechnology, Fatima Jinnah Women University, Rawalpindi, Pakistan; ^2^ Department of Biotechnology, University of Kotli Azad Jammu and Kashmir, Kotli, Pakistan

**Keywords:** biosurfactant production, secondary metabolites biosynthesis, oil remediation, lipopeptides, fengycin, surfactin

## Abstract

Biosurfactants are a diverse group of amphiphilic compounds obtained from microbes. In the present study, the genomic analysis of biosurfactant-producing *Bacillus subtilis* MB415 and MB418 obtained from oil-contaminated soil was performed. Initially, the strains were screened for biosurfactant production by hemolytic assay, emulsification index, and oil displacement. Further FTIR analysis of extracted biosurfactants revealed the presence of lipopeptides. The sequenced genomes of MB415 and MB418 were of 4.2 Mbps with 43% GC content. Among more than 4,500 protein-coding genes, many were involved in virulence, metal/multidrug resistances, flagella assembly, chemotactic response, and aromatic ring hydroxylating dioxygenases. An annotation analysis revealed that both genomes possessed non-ribosomal synthetase gene clusters for the lipopeptide synthetases *srf* and *fen* responsible for surfactin and fengycin production. Comparative studies of both genomes highlighted variability in gene operons mainly for surfactin biosynthesis.

## Introduction

Natural products comprise a variety of bioactive compounds like bacteriocins, terpenoids, antibiotics, and biosurfactants (Raaijmakers et al., 2010; Anuradha, 2010). Among these, biosurfactants are surface metabolites produced by microbes during the stationary phase of growth. The broad spectrum of structurally different biosurfactants, generally glycolipids, phospholipids, lipopeptides, neutral lipids, and fatty acids, consist of hydrophobic moiety (fatty acids) of varying lengths linked to hydrophilic peptide chains of 7–10 amino acids (Geys et al., 2014). Lipopeptides are one of the major classes of lower molecular weight biosurfactants produced by microorganisms. Around 90 compounds from 26 different lipopeptide families have been identified in the last two decades (Liu et al., 2015). Microbial biosurfactants have gained special interest nowadays because of their surface and interfacial properties, eco-friendly characters, and diverse applications in industrial, health, and environmental sectors. For example, microbial glycoconjugates enhance degradation of toxic organic pollutants by increasing their bioavailability by lowering surface tension and producing a solvent interface (Bhatt et al., 2021).

Biosurfactants are encoded by several genes and synthesized by a variety of microorganisms such as *Bacillus* sp., *Pseudomonas* sp., *Acinetobacter* sp., *Candida* sp., *Cryptococcus* sp., *Penicillium* sp., *Aspergillus* sp., *Kurtzmanomyces* sp., *Rhodococcus* sp., *Sphingomonas* sp., *Arthrobacter* sp., *Lactococcus* sp., and *Pseudozyma* sp. (Borah et al., 2021). Among these, the genus *Bacillus* is the most prevalent in biosurfactant production. Carolin et al. (2021) reported on the lipopeptide biosurfactant production by *Bacillus* sp., and its significant role in the degradation of aromatic amine 4-chloroaniline compound. Similarly, biosurfactants also exhibit a promising potential to efficiently eliminate toxic heavy metal pollutants by using multiple biosurfactant-metal binding stratagems, that is, emulsification, mobilization, complexation, and solubilization (Mishra et al., 2021).

Lipopeptides are biosurfactants mainly comprise a hydrophilic peptide ring and hydrophobic fatty acid moieties. Based on their structure, they are further characterized into cyclic lipopeptides (CLPs) and linear lipopeptides. The cyclic lipopeptides, commonly produced by *Bacillus subtilis,* include fengycin, surfactin, and iturin. They are composed of a peptide ring of 7 or 10 amino acids with a long hydrophobic fatty acid chain. The fatty acid chain lengths vary in each type, that is, surfactin has a chain length of C_13_–C_16_, while iturin’s chain is C_14_–C_17_, and that of fengycin is C_14_–C_18_ (Hu et al., 2019).

Surfactin has many congeners because of the variation in the length of the fatty acid chain and the types of amino acids. The structure of surfactin offers diverse applications in several global issues such as medicine and environmental protection (Gudiña et al., 2013). It was discovered as a potent fibrin clot-inhibitor and as an antibacterial and antiviral agent. It also showed hypocholesterolemic and anti-tumor activities. Owing to its remarkable surface-, interface-, and membrane-active action, it has the ability to cross plasma membrane barriers and viral envelopes (Santos et al., 2018). It can lower the surface tension of water from 72 to 27 mN/m and is highly thermally stable and salt tolerant. Because of these properties, it possesses huge potential in the microbial enhancement of oil recovery (MEOR) and considered a good candidate for the bioremediation of contaminated soils and sub-surface environments (Hu et al., 2019).

Fengycin is another lipopeptide consisting of a decapeptide linked to a saturated or unsaturated fatty acid (C14–C18). The ring structure of its cyclic peptide is formed by linking the residue at position 3 to the C-terminal–COOH group of the amino acid at position 10 (Fanaei et al., 2021). Fengycins are predominantly produced by *Bacillus* spp. with potential bioactivities. They are active against phytopathogenic fungi and bind with their plasma membrane, thereby causing cell lysis and leakage (Gimenez et al., 2021).

The biosynthesis of these lipopeptides is governed by a complex set of proteins called non-ribosomal peptide synthetases (Wang et al., 2014). Gene clusters for the synthesis of such compounds have been found in the *Bacillus* genera (Donio et al., 2013). Several structurally and functionally diverse molecules need to be screened and characterized to create a better understanding of such biomolecules (Lu et al., 2014; Aleti et al., 2015; Singh and Tiwary, 2016).

Next-generation sequencing has revolutionized the discovery of natural products, chemicals, and biosynthetic enzymes greatly used in the fields of biotechnology and biomedicine (Luo et al., 2014). Biosurfactant production in various *Bacillus* species has been widely reported in many studies. Moreover, many *Bacillus* species are known for their metabolic capability, environmental versatility, and their ability to remove xenobiotic compounds and heavy metals via biosurfactant production (Arora, 2020). However, only limited literature is available on the genetic basis of biosurfactant production in bacteria. To this end, our research is focused on the whole genome sequencing and analysis of biosurfactant-producing and hydrocarbon-degrading indigenous *Bacillus subtilis* strains MB415 and MB418 isolated from hydrocarbon-contaminated soil. The genome analysis of both strains also indicated the gene clusters for surfactin and fengycin lipopeptides.

## Materials and Methods

### Isolation and Characterization of Biosurfactant-Producing Bacteria

Soil samples were collected from the oil-contaminated site of Missa Kiswal oil field, Gujar Khan, Pakistan. Bacterial strains were isolated on Bushnell Hass mineral salt medium (Bushnell and Haas, 1941) by the spread plate method. Isolated bacterial strains were screened for biosurfactant production by a blood hemolysis assay (Mulligan et al*.,* 1984). For this purpose, 5% defibrinated goat blood was added to the blood agar base. The emulsification activity (Cooper and Goldenberg, 1987) was determined by taking 2 ml of kerosene with an equal volume of the culture supernatant and vortexed vigorously for a few minutes. The emulsion formed was allowed to be stable for 24 h and the emulsification index was calculated as the percentage of the height of the emulsion layer (mm) divided by the total height of the liquid (mm). Furthermore, the oil-spreading technique and surface tension of culture supernatants were measured by using a torsion balance (Lin and Timasheff, 1996; Morikawa et al., 2000). Extraction and purification of biosurfactants from selected isolates were done by following the protocol of Qiao and Shao (2010). The extracted biosurfactants were analyzed through FTIR spectroscopy.

### Whole Genome Sequencing

For molecular characterization, isolates were sequenced for 16S rRNAs that are submitted to NCBI. In addition, the whole genome sequence of both strains was performed to get an insight into the genetic composition. Cells were grown in Luria-Bertani (LB) broth at room temperature. The genomic DNA was extracted in the late exponential phase. The cells were collected by centrifugation at 4°C and 10,000 g for 10 min. The supernatant was discarded and the cells were suspended in 200 µL of PBS. The samples were then treated with lysozyme (1.33 mg/ml) and incubated for 5 min. Proteinase K (20 µL) was added to the cells that were incubated for 1 hour at 37°C. After this, 200 µL of buffer AL was added to the samples, mixed thoroughly, and kept at 56°C for 10 min. Furthermore, 96–100% ethanol (200 µL) was used for washing nucleic acid pellets. The mixture collected was centrifuged at 8,000 rpm for 1 min and the flow-through was discarded. Buffer AW2 (500 µL) was added to the spin column, centrifuged at 14,000 rpm, and the flow-through was discarded. Finally, the DNA was eluted with an elution buffer AE (200 µL) that was incubated for 1 min at room temperature and centrifuged at 6,000 rpm for 1 min to collect the DNA in a sterile Eppendorf tube (Aslam et al., 2018). The concentration and purity of DNA were determined by using a nanodrop spectrophotometer ND-1000 (Nanodrop Technology, Wilmington, DE). The qualitative assessment was done by visualizing genomic DNA on 1% agarose gel stained with ethidium bromide. The genomic DNA of both strains MB415 and MB418 were sequenced using the Illumina MiSeq (2 × 300 bp) platform.

### Sequence Assembly and Annotation

The obtained sequence reads were assembled through SPAdes 3.1 including error correction, deBruijn graph assembly for arrangement into contigs and their scaffolding (Bankevich et al., 2012). The quality of the assembled sequence data was assessed using Quast (Gurevich et al., 2013). Finally, the genomic data were annotated through different annotation servers.

Gene prediction and functional annotation analysis was performed using three different pipelines: Integrated Microbial Genomes expert review (http://img.jgi.doe.gov), Rapid Annotation Subsystem Technology (Aziz et al., 2008), and Genome Annotation Pipeline (PGAP) by NCBI (http://www.ncbi.nlm.nih.gov/genome/annotation_prok). The genes were identified by Prodigal (Hyatt et al., 2010). The predicted CDSs were translated and searched against NCBI, non-redundant databases, UniProt, TIGRfam, Pfam, PRIAM, COG, KEGG, and interPro databases performed by the IMG ER platform that is developed by the Joint Genome Institute, Walnut Creek, CA, United States (Markowitz et al., 2009). In addition, transfer RNAs and rRNAs were identified using tRNAscan-SE (Lowe and Eddy, 1997) and RNAmmer (Lagesen et al., 2007).

## Results

### Screening of Biosurfactant-Producing Bacteria

Fifteen hydrocarbon-degrading bacteria were isolated on Bushnell Haas Mineral salt medium from oil-polluted soil samples. Isolates with distinct morphological characteristics and competent to utilize different hydrocarbon substrates as a sole carbon source were selected, purified, and screened for biosurfactant production. Multiple screening assays were performed for the identification of potential biosurfactant producers, that is, oil spreading assay, emulsification index, and surface tension and hemolytic assays. Two isolates, that is, MB415 and MB418 showed a remarkable reduction in surface tension values, that is, 20 and 35 dyn/cm, respectively. Both strains showed distinct oil displacement and low emulsification index. Strain MB418 exhibited complete, while MB415 displayed partial hemolysis of erythrocyte cells (Table 1).

**TABLE 1:** Results of biosurfactant screening assays.

Bacterial isolates	Hemolysis	Oil spreading	Emulsification index (%)n	Surface tension (dynes/cm)
*Bacillus subtilis* MB415	Α	+ (10s)	20 ± 2	20 ± 0.5
*Bacillus subtilis* MB418	Β	+(2min)	15 ± 2.5	35 ± 5

*α* = incomplete hemolysis *β* = complete hemolysis.

**FIGURE 1:**
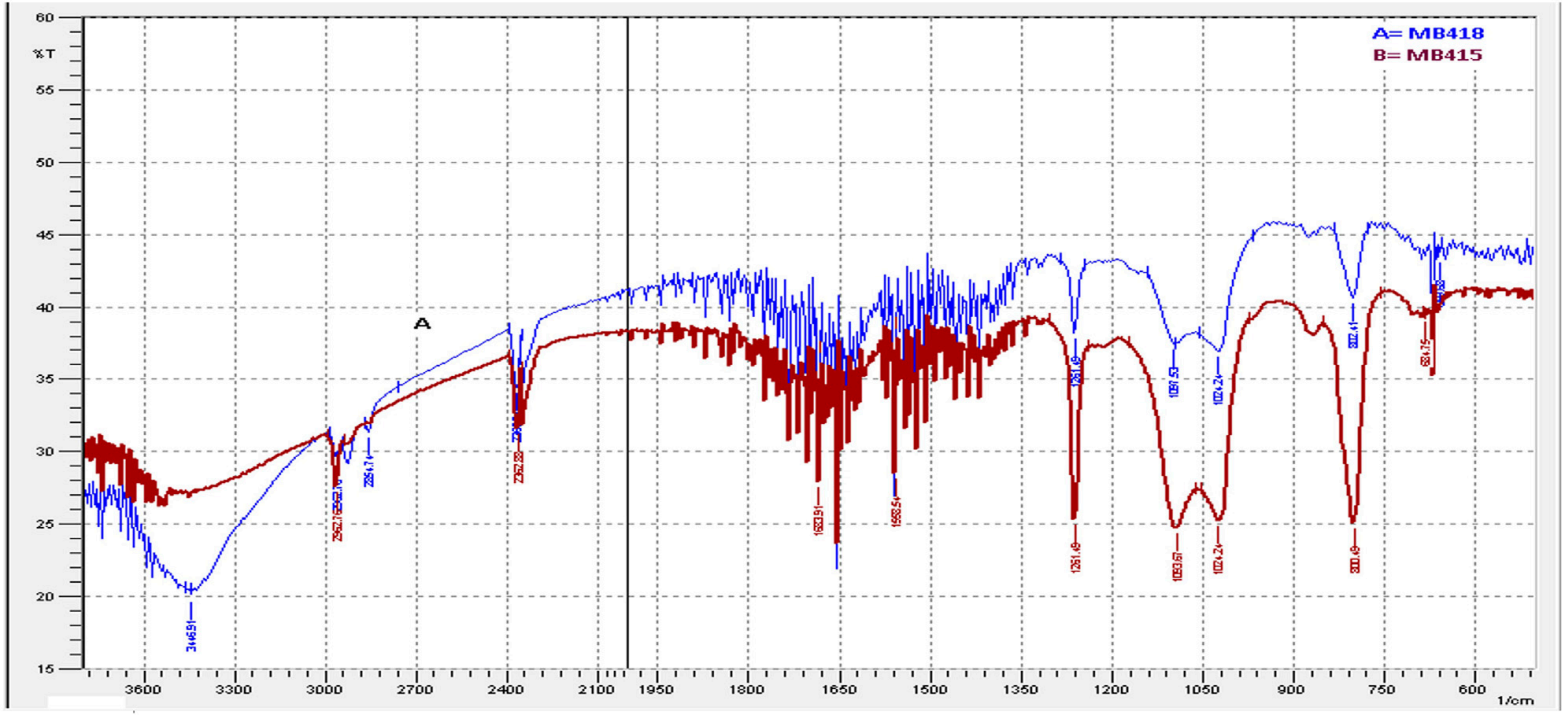
FTIR spectra of extracted biosurfactants.

In the present study, the maximum emulsification activity of diesel was recorded by culture supernatants of bacterial strains. The emulsification ability is attributed to the augmented biodegradation potential of petroleum hydrocarbons. The FTIR spectrum strongly reflects that the extracted compound was a potential biosurfactant with peptide and aliphatic hydrocarbon moieties. Significant absorbance peaks of the biosurfactant isolated from *B. subtilis* MB415 and MB418 observed C-O, C-H, and C=O in the regions of 1,000–1,320/cm, 2,850–3,000/cm, and 1,665–1760/cm stretching mode, respectively, verifying the presence of glycolipids (Figure 1).

### Genome Sequencing

The genomes of *B. subtilis* strains MB415 and MB418 were sequenced to identify their hydrocarbon degradation potential and applications in bioremediation. The genome project was deposited in the Integrated Microbial Genome (IMG ER) online database and the Genome Online Database (GOLD) (Pagani et al., 2012). The whole-genome shotgun (WGS) project of both isolates was deposited at DDBJ/EMBL/GenBank under accession numbers LYDW00000000 and MQSR00000000 for *B. subtilis* MB418 and MB415, accordingly.

### Genome Properties

The genome sequences of *B. subtilis* MB415 and MB418 were generated with the help of Illumina Miseq sequenced data. For both the genomes, the paired end reads 4, 120, 685 bp and 4, 266, 089 bp respectively, were assembled into contigs and scaffolds. Genome statistics according to the NCBI Prokaryotic Genome Annotation Pipeline are summarized in Table 2.

**TABLE 2:** Genome characteristics of *Bacillus subtilis* MB415 and *Bacillus subtilis* MB418.

Strain	Accession number	Size Mbps	GC content	Number			
				Contigs	Subsystems	Cds	RNA
MB415	MQSR00000000	4267093	43.1	246	479	4,610	116
MB418	LYDW00000000	4118930	43.6	289	479	4,455	121

### Annotation Analysis and Subsystem Features

The draft genome of *B. subtilis* MB418 contains 4,543 genes containing 4,055 genes with coding sequences and 362 pseudogenes. The genome was found to have 126 RNA genes including 85 tRNAs and 35 rRNAs comprising 11 genes for 5S, 12 each for 16S and 23S rRNAs, while six were non-coding RNAs identified through RNAmmer and tRNA Scan S.E. The *B. subtilis* MB415 genome of 4, 267, 672 bps contains 4,812 genes with 4, 416 protein-coding genes and 121 rRNAs including 86 genes for tRNAs and 11, 12, 7 genes carrying codes for 5S, 16S, and 23S respectively.

**FIGURE 2:**
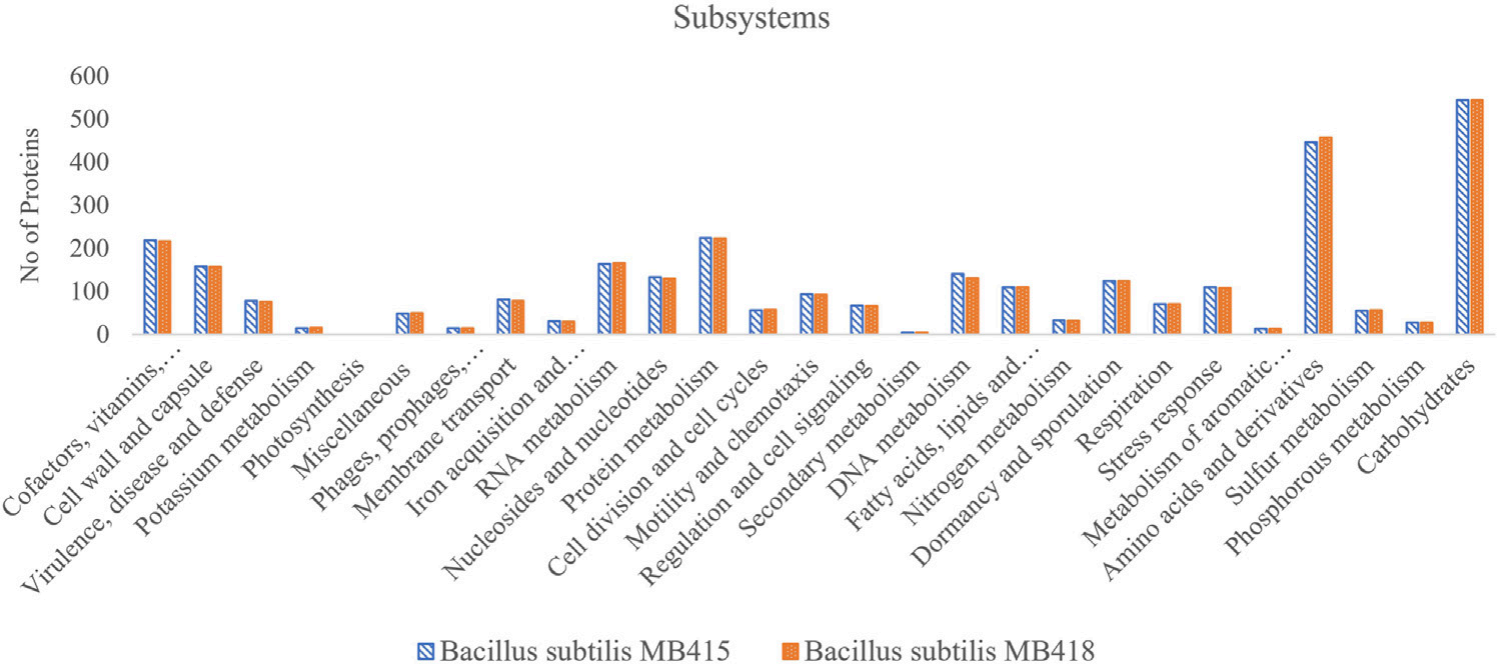
Subsystem features of the two *Bacillus subtilis* strains (MB415 and MB418).

The annotation of the genome *B. subtilis* MB418 using the RAST server (Aziz et al., 2008) identified genes involved in the tolerance against metals, that is, arsenic (6 genes), copper (4 genes), and Co/Zn/Cd (4 genes). In addition, the genome has a multidrug resistance operon mdtRP mostly present in the genus *Bacillus*, and beta-lactamase, streptothricin, and vancomycin which are mostly found in gram-positive bacteria. Among 479 subsystems, 545 genes were specified for carbohydrates and 458 for amino acids and their derivatives. The genome also possesses a significant number of genes allocated for chemotaxis (15 genes) and flagellar motility (78 genes). There were around 117 genes which confer proteins for dormancy, sporulation-like genes designated for biofilm matrix protein component TsaA, sporulation gene operons, spore germination, hydrolysis, and sporulation cluster IIIA and cluster II, while in *B. subtilis* MB415, many genes involved in metal tolerance including arsenic (6), copper (5), and Co/Zn/Cd resistance (4) were identified. However, few genes were found to be involved in antibiotic resistance among multidrug resistance operon efflux pumps (8) and beta-lactamase (3), along with genes for vancomycin, streptothricin, and fosfomycin resistance. Both genomes possess genes for secondary metabolite biosynthesis that includes surfactin, fengycin, and siderophore. In addition, genes encoding enzymes involved in different aromatic compound metabolisms, that is, peripheral anaerobic and central aromatic intermediates formed during degradation were also predicted in both genomes (Figure 2).

### Biosurfactant-Producing Genes

Emerging next-generation sequencing technology coupled with other computational efforts of annotation has enabled us to look into several potential genes simultaneously. This has improved our understanding of essential metabolic pathways and the adaptive variation of organisms in stressed environments. Similarly, bacterial diversity inhabiting soil with a high concentration of hydrocarbons (such as soil in and around oil fields) possessed certain genes for biosurfactant (surfactin, fengycin, and iturins) production.

Annotation results revealed that both genomes MB415 and MB418 carry operons responsible for the biosynthesis of secondary metabolites such as surfactin and fengycin. In *B. subtilis* MB418, surfactin-producing genes including *srf*AA codes for the surfactin family lipopeptide synthetase-A. This protein belonged to the COG1020 category of clusters of orthologous groups (COG) for the conserved protein domain family *EntF* called non-ribosomal peptide synthetase component F. In addition, it showed a close relationship with the phosphopantetheine attachment site protein (IPR006162) and acyl carrier protein of super family SSF47336 (IPR009081). It also shares similarities with the peptide chains of IPR020806 polyketide synthase, phosphopantetheine-binding domain, and others including IPR020845, SSF56801, and TIGR01720 of the non-ribosomal peptide synthase domain. It was relevant to many other protein domains including PP-binding, condensation, AMP binding_C, and pfam13745-HXXPF_rpt. Next to this is the gene *srf*AC encode for the protein surfactin family lipopeptide synthetase C. This gene of 3,828 bp is translated into a protein of 1,275 amino acids, a non-ribosomal peptide synthetase component F. This protein belongs to the phosphopantetheine attachment site (IPR006162) and other protein classes of databases InterPro, pfam, and TIGRfam like *srf*AA.

The genome also possesses gene *srf*AD (729 bp) encodes for the thioesterase domain-containing protein comprising 242 amino acids. According to clusters of orthologous groups, it belongs to the category COG3208 surfactin synthase thioesterase subunit exhibiting 91.32% similarity with alpha/beta-hydrolases of pfam00975 and SSF53474. Lastly, the gene for ycxA similar to COG2271 called sugar-phosphate permease comprises 1,227 bp and encodes for 408 amino acid sequences. The genome encodes a permease of the major facilitator superfamily (KOG2533) related to the transporter class TC: 2. A. 1 of IPR016196, IPR020846 with 85.78% alignment with pfam07690.

**FIGURE 3:**
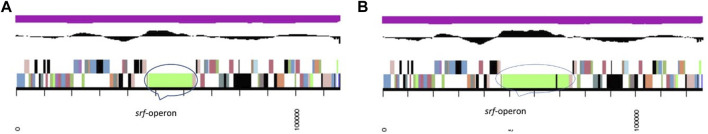
Portion of chromosomal map having scaffold with *srf*-operon **(A)** MB418 and **(B)** MB415. From outside to the center: genes on the forward strand (color by COG categories), genes on the reverse strand (color by COG categories), RNA genes (tRNAs green, rRNAs red, other RNAs black) GC content GC skew.

This gene arrangement was different in *B. subtilis* MB415. The operon starts with the gene *srf*AA of 10,764 bp encode for the surfactin family lipopeptide synthetase A (COG1020). The acyl-CoA carrier protein, also called surfactin family lipopeptide synthetase B gene *srf*AB (3329bp), was identified in the genome of *B. subtilis* MB415. This protein of 3,329 amino acids has a maximum similarity with IPR009081 (acyl carrier protein-like), IPR020845 (AMP-binding, conserved site), and SSF52777 (CoA-dependent acyltransferases). Next to this is the gene for the condensation protein SrfAB of pfam00668. In addition, it also possesses the gene *srfAC* of 3828bp, *srfAD* thioesterase domain-containing protein, and ycxA-sugar phosphate permease. The arrangement of top COG hits on the scaffold with *srf*-operon in the genome of *B. subtilis* MB418 and MB415 (Figure 3).

**FIGURE 4:**
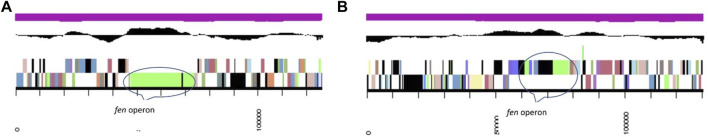
Fengycin synthetase gene arrangement on respective scaffolds **(A)** MB418 and **(B)** MB415. From outside to the center: genes on the forward strand (color by COG categories), genes on the reverse strand (color by COG categories), RNA genes (tRNAs green, rRNAs red, other RNAs black) GC content GC skew.

Fengycin produced by various *Bacillus* strains is expected to form a lactone between the hydroxyl group of L-Tyr3 and C-terminal carboxyl group L-II-e and fengycin synthase (FenC, FenA, and FenB). In the MB418 genome, the gene *fen*C encoding fengycin family lipopeptide synthetase A (KO: K15664 ppsA) was identified (Figure 4). However, in case of the *Bacillus subtilis* MB415 genome, the operon of fengycin starts with the gene *fen*B, comprising 243 bps and belonging to fengycin lipopeptide synthetase E (KO: K15668). It contains the non-ribosomal synthetase component F of COG1020. This protein of 2,042 amino acids consists of fengycin family lipopeptide synthetase A (KO: K15664). In between these genes, a gene for the non-ribosomal peptide synthase and condensation domain-containing protein, amino-acid adenylation domain, AMP-binding enzyme C-terminal domain, and an uncharacterized lipoprotein *yddw* were identified.

## Discussion

In the present study, we investigated the biosurfactant production from the hydrocarbon degraders of the Missa Kiswal oilfield. Two *B. subtilis* strains were able to produce lipopeptide biosurfactants. Similarly, a study reported the strain *B. amyloliquefaciens* An6 as a potent biosurfactant producer with diesel oil degradation efficiency (Ayed et al., 2015). Hydrocarbon-degrading bacteria possess an innate potential to produce biosurfactants which aid them in the bioavailability of hydrocarbon substrates (Antoniou et al., 2015).

The genomes of the strains isolated in the present study also decipher *Srf* operons responsible for the biosynthesis of surfactin and fengycins. In the case of *B. subtilis,* mostly fengycin and surfactin have been reported in many studies (Pereira et al., 2013). Jadeja et al. (2019) reported the *sfp* and NPRS gene in *Bacillus* sp. AKBS9 and emulsan biosynthetic gene cluster in *Acinetobactor* sp. AKBS16 for biosurfactant production through the whole genome sequence analysis of the aforementioned strains.

*B. subtilis* is one of the predominant lipopeptide cell factories (Steller et al., 1999). The genetic makeup of biosurfactant-producing organisms is one of the major factors governing the biosynthesis of biosurfactants. Different molecular studies demonstrated the metabolic pathways, operons, and enzymes for the extracellular production of many biosurfactants. Wang et al. (2022) identified entire core synthetic genes for bacillibactin, fengycin, and surfactin production in a novel facultative-halophilic, long-chain hydrocarbon degrader, that is, *Bacillus* sp. XT-2. Surfactin is a cyclic lipopeptide produced non-ribosomally by surfactin synthetase, a multi-enzyme peptide synthetase complex. The biosynthesis of other lipopeptides is also mediated by similar enzyme complexes. Non-ribosomal peptide synthetases (NRPSs) involved in lipopeptide biosynthesis exhibit a high degree of similarity in structural conformation even in highly distant microbial species (Dhasayan et al., 2015). Fengycin is another cyclic lipodecapeptide containing four d-amino acids and ornithine in the peptide chain. Fengycin looks like a mixture of isoforms that show differences in length and branching of the *β*-hydroxy fatty acid, and the amino-acid composition of the peptide ring (Hu et al., 2007).

The genomes presented in our study decrypt the *B. subtilis* sequence variation and detailed comparisons of this vital model species. Comparisons of whole-genome sequences of different bacteria revealed significant genomic variability among phylogenetically interrelated bacterial species. Even sequences with 100% homology exhibit low conservation in the total gene content. *B. subtilis* is one of the most extensively studied spore forming, non-pathogenic, gram-positive bacteria ubiquitously found in soil (Earl et al., 2007; Chen et al., 2013). Another study demonstrated the complete genome sequence analysis of polystyrene-degrading deep sea *B. paralicheniformis* G1 strain comprising 4,281,959 bp with 45.88% GC content and encoded 4,213 protein-coding genes. Numerous genes encoding monooxygenase, dioxygenase, peroxidase, esterase, and hydrolase involved in the degradation of synthetic polymers were identified along with the genes associated with flagellum-dependent motility, chemotaxis, biofilm formation, and siderophores biosynthesis (Kumar et al., 2021).

**FIGURE 5:**
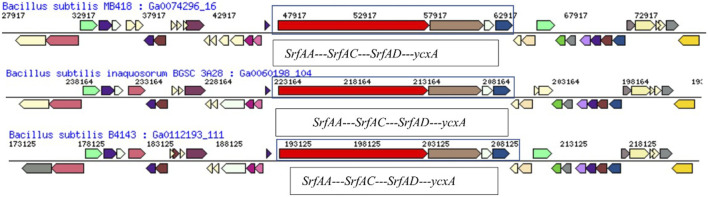
Comparison of the hydrocarbon-degrading gene locus *srf* operon of *Bacillus subtilis* MB418 with other *Bacillus* species.

The genetic regulation of lipopeptides is governed by the four open reading frames (ORFs) in the *srfA* operon directing the surfactin synthesis including *srfAA*, *srfAB*, *srfAC,* and *srfAD* (Zhu et al., 2021). Surfactin is synthesized by a multi-enzyme synthetase complex mainly comprising *SrfA, SrfB,* and *SrfD* subunits. *Srf* operon for biosurfactant pathways consists of the gene *srfAA, -AB*, -*AC,* and*–AD* in *Bacillus* spp. The annotation analysis of the genome *B. subtilis* MB415 and MB418 revealed that both strains also possess this operon (Figure 5). According to previous studies, in the *Bacillus subtilis* species, the synthesis of surfactin is based on three amino-acid activating components of surfactin synthetase (*SrfA, -B,* and *-C*) that are activated by *SrfD*. The gene *srf*AD also possesses thioesterase that is required for the last amino acid in the growing peptide chain that results in the cyclic structure of the biosurfactant and *B. subtilis* (Vedaraman and Venkatesh, 2011).

**FIGURE 6:**
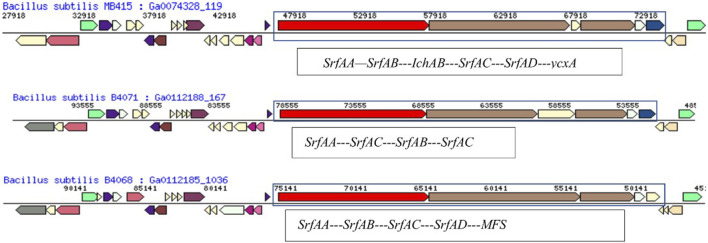
Comparison of the hydrocarbon-degrading gene locus *srf* operon of *Bacillus subtilis* MB415 and other *Bacillus* species.

A comparative genome analysis showed gene arrangements of surfactin-producing operon in the draft genome of *B. subtilis* MB418 shared its neighborhood region showing the same top COG hits with *Bacillus subtilis inaquosorum* BGSC 3A28 and *B. subtilis* B4143. With the difference in the SrfAA protein, in *Bacillus subtilis* MB418 there are 3,586 amino-acid sequences, while it is 3,587 amino acids in the rest of the two closely related genomes. However, in the case of MB415, the operon is entirely different with genes encoding for more proteins and a different arrangement as shown in Figure 6.

The gene *srfAB* encodes for surfactin family lipopeptide synthetase AB and consists of 3,329 amino acids. A comparison of the gene neighborhood showed maximum similarity with *B. subtilis* B4071 (Berendsen et al., 2016) having the gene for the protein SrfAB (2695aa) (Figure 6). With the exception, *B. subtilis* MB415 had an additional gene for non-ribosomal peptide synthase not present in the genome of B4071. The gene operon in the genome of MB415 ends at gene (*ycxA*) for sugar-phosphate permease, while in the *B. subtilis* MB4068 (Berendsen et al., 2016) operon, this gene is replaced with another gene for the major facilitator superfamily (Figure 6).

Furthermore, genomes which carry genes for the same proteins vary in sequence length than the ones present in the draft genome of MB415. For example, the *srf*AA gene of *B. subtilis* MB415 is of 10,764 bp codes for 3,587 amino acids*.* However, this gene sequence is relatively small in the previously reported surfactant-producing *B. safensis* CCMA-560 and *B. amyloliqueficiens* comprising 10, 713bps (3570aa) and 10, 701bps (3566aa), respectively. The gene *srf*AC (3828bp) sequence is the same in the genomes of *B. subtilis* MB418 and MB415 and in the rest of the *Bacillus* species. The gene encoding thioesterase in the genomes of both *B. subtilis* MB415 and MB418 comprises 3,834 bps while in *B. pumilus*, it is encoded by the *srf*AD gene (732bp) (Jiang et al., 2016). Similarly, surfactin produced by *B. subtilis* strains isolated from soil consists of seven amino acids and 13–15 lipid chains forming a cyclic lipopeptide as described previously by researchers (Sekhon et al., 2011; Sachdev and Cameotra, 2013).

The genome analysis of both isolates unveiled potential gene operons for the biosynthesis of a secondary metabolite, fengycin. Fengycin, also called plipastatin, is an anti-fungal antibiotic that inhibits filamentous fungi but is ineffective against yeast and bacteria (Volpon et al., 2000). It also has the potential to inhibit phospholipase and biofilm formation in many bacteria (Thaniyavarn et al., 2003). Fengycin is a type of lipodecapeptide with moderate surfactant activity normally produced by several strains of *Bacillus* spp. *Fen* operon comprises an N-acyl domain at the N-terminus of *FenC* and five non-ribosomal peptide synthetase subunits assembled in a co-linear chain in the following order: *Fen*C, *Fen*D, *Fen*E, *Fen*A, and *Fen*B (Guo and Yu, 2014).

**FIGURE 7:**
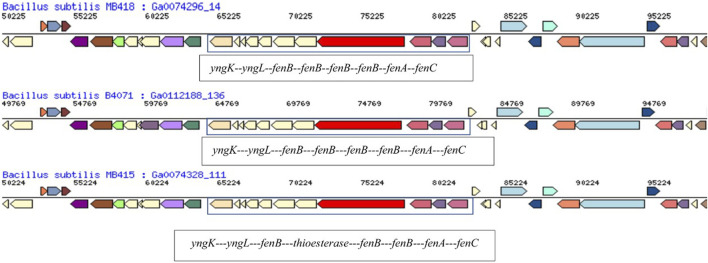
Comparison of the hydrocarbon-degrading gene locus *fen* operon of *Bacillus subtilis* MB415, MB418, and other *Bacillus* species.

The fengycin synthetase gene operon in the draft genome of *B. subtilis* MB418 exhibited maximum similarity with *B. subtilis* B4071. However, in the genome of *B. subtilis* MB415, an esterase-producing gene is identified instead of *fenB* for fengycin lipopeptide synthetase E as indicated in other closely related *Bacillus* species (Figure 7). A recent study also reported three genes related to the gene cluster responsible for fengycin biosynthesis (*fenBCD*) in *B. velezensis* and *B. siamensis* (Zeng et al., 2021). Similarly, in another study, five ORFs, namely, *Fen*A, *Fen*B, *Fen*C, *Fen*D, and *Fen*E, were identified for fengycin production in the *Bacillus velezensis* strain P45 (da Rosa et al., 2022).

## Conclusion

Indigenous bacterial strains inhabiting a polluted environment acquire tremendous potential to utilize petroleum hydrocarbons than non-native microbes. The present study demonstrated the production of lipopeptide biosurfactants from *B. subtilis* MB415 and MB418. The genome analysis of both *B. subtilis* isolates MB415 and MB418 contain a gene locus that enhanced their proficiency in degrading oil compounds. The presence of genes for flagella and secondary metabolite biosynthesis supported the experimental outcomes of emulsification and oil utilization capacities of both strains.

## Data Availability

The datasets presented in this study can be found in online repositories. The names of the repository/repositories and accession number(s) can be found at: https://www.ncbi.nlm.nih.gov/genbank/, LYDW00000000, https://www.ncbi.nlm.nih.gov/genbank/, MQSR00000000.
